# Singularity of a relativistic vortex beam and proper relativistic observables

**DOI:** 10.1038/s41598-020-64168-0

**Published:** 2020-05-04

**Authors:** Yeong Deok Han, Taeseung Choi, Sam Young Cho

**Affiliations:** 1 Department of Computer Science and Engineering, Woosuk University, Jincheon, Chungbuk 27841 Korea; 2 Institute of General Education, Seoul Women’s University, Seoul, 01797 Korea; 3 School of Computational Sciences, Korea Institute for Advanced Study, Seoul, 02455 Korea; 4 Centre for Modern Physics and Department of Physics, Chongqing University, Chongqing, 400044 China

**Keywords:** Theoretical particle physics, Matter waves and particle beams

## Abstract

We have studied the phase singularity of relativistic vortex beams for two sets of relativistic operators using circulation. One set includes new spin and orbital angular momentum (OAM) operators, which are derived from the parity-extended Poincaré group, and the other set consists of the (usual) Dirac spin and OAM operators. The first set predicts the same singularity in the circulation as in the case of nonrelativistic vortex beams. On the other hand, the second set anticipates that the singularity of the circulation is spin-orientation-dependent and can disappear, especially for a relativistic paraxial electron beam with spin parallel to the propagating direction. These contradistinctive predictions suggest that a relativistic electron beam experiment with spin-polarized electrons could for the first time answer a long-standing fundamental question, i.e., what are the proper relativistic observables, raised from the beginning of relativistic quantum mechanics following the discovery of the Dirac equation.

## Introduction

Nonrelativistic electron vortex beams carrying orbital angular momentum (OAM) have recently been studied and are well-understood using the paraxial approximation of the Schrödinger equation^1–6^. The wavefunction of a nonrelativistic electron vortex includes a phase singularity factor, ${e}^{il\phi }$, where $\phi$ is the azimuthal angle around the axis of the vortex, and the electron vortex beam can carry orbital angular momentum of $l\hslash$ in which $l$ is an integer known as the topological charge^1^. As the energy of electron vortex beams reaches the relativistic regime of 200~300 keV^1,2,7,8^, the validity of the interpretation of the rotational motion of the high-energy electrons in the experiment as a relativistic electron vortex is questioned^9–15^.

To understand such relativistic electrons, one should use the Dirac equation^16^, which successfully describes relativistic electrons, instead of the Schrödinger equation. However, in the usual Dirac theory, the spin angular momentum and orbital angular momentum of an electron are not separately conserved unlike in the Schrödinger theory. As a result, Bialynicki-Birula *et al*. proved the assertion that any acceptable solutions of the Dirac equation cannot be eigenstates of the (usual) Dirac OAM, and showed that the vortex lines continuously smeared out into all space for their exponential solutions, which become the standard vortex wavefunction in the nonrelativistic limit^12^. This raised the question of whether a relativistic vortex can be generated from high-energy electron beams. In contrast, Barnett^13^ argued that relativistic electron vortices with a well-defined OAM and phase singularity truly exist using the so-called Foldy-Woutheysen (FW) representation^17^ in which the vortex charge is related to the eigenvalues of OAM, as is the case for nonrelativistic vortices.

The controversal results of Bialynicki-Birula *et al*. and Barnett^12,13^ originated from the use of different spin and OAM operators as relativistic operators, as indicated by Bliokh *et al*.^18^. Explicitly, Bialynicki-Birula *et al*. used the usual Dirac spin and OAM operators, but Barnett used the FW mean spin and the FW mean OAM operators. Essentially, such other choices are due to a lack of understanding of relativistic operators. As an unsolved fundamental issue, obtaining a proper relativistic spin operator for massive spin-1/2 particles has been a long-standing problem from the beginning of relativistic quantum mechanics^17,19–27^. Many discussions have attempted to suggest possible proper description of spin for massive elementary particles.

Recently, two of the present authors, i.e., Choi and Cho^28^ rigorously derived a spin operator for the Dirac field that transforms covariantly under the Lorentz transformation. We call this spin operator the new spin operator to distinguish it from the other spin operators such as the Dirac and the FW mean spin operators. The new spin operator was shown to be the generator of the *SU*(2) little group of the Poincaré group and admits a representation of the Poincaré group extended by the parity (space inversion) in which the Dirac spinor resides. Additionally, the Lorentz boost represented by the new spin operator can provide a representation of the parity operator from which the covariant Dirac equation is derived. In view of theoretical consistency and completeness, the new spin operator representing the parity-extended Poincaré group is compelling as a proper relativistic spin operator. Furthermore, based on the consistent relativistic description of massive particles, the new spin operator can be effectively defined as a particle spin operator and an antiparticle spin operator according to the action of the spin on the particle state and the antiparticle state, respectively (Table 1). Straightforwardly, the particle spin operator was also shown to be equal to the FW mean spin operator^28^.

**Table 1:** Table of the properties, operator-representatives, and relations of the new spin **S**_*N*_, the particle spin **S**_*P*_, and the antiparticle spin **S**_*AP*_.

$${{\bf{S}}}_{{\boldsymbol{N}}}=\frac{{\boldsymbol{E}}}{{\boldsymbol{m}}}{{\bf{S}}}_{{\boldsymbol{D}}}-\frac{{\bf{p}}({{\bf{S}}}_{{\boldsymbol{D}}}\cdot {\bf{p}})}{{\boldsymbol{m}}({\boldsymbol{E}}+{\boldsymbol{m}})}+{\boldsymbol{i}}{{\boldsymbol{\gamma }}}^{{\bf{5}}}\frac{{\bf{1}}}{{\boldsymbol{m}}}({{\bf{S}}}_{{\boldsymbol{D}}}\times {\bf{p}}).$$	
Particle	Antiparticle
${H}_{D}=\alpha \cdot {\bf{p}}+\beta m$	${\tilde{H}}_{D}=-\,\alpha \cdot {\bf{p}}+\beta m$
${{\bf{S}}}_{N}{\psi }_{P}({p}^{\mu })={{\bf{S}}}_{P}{\psi }_{P}({p}^{\mu })$	${{\bf{S}}}_{N}{\psi }_{AP}({p}^{\mu })={{\bf{S}}}_{AP}{\psi }_{AP}({p}^{\mu })$
${{\bf{S}}}_{P}={{\bf{S}}}_{D}+\frac{{\bf{p}}({\bf{p}}\cdot {{\bf{S}}}_{D})-{\bf{p}}\cdot {\bf{p}}{{\bf{S}}}_{D}}{E(E+m)}+i\beta \frac{({\bf{p}}\times \alpha )}{E}$	${{\bf{S}}}_{AP}={{\bf{S}}}_{D}+\frac{{\bf{p}}({\bf{p}}\cdot {{\bf{S}}}_{D})-{\bf{p}}\cdot {\bf{p}}{{\bf{S}}}_{D}}{E(E+m)}-i\beta \frac{({\bf{p}}\times \alpha )}{E}$

Detailed explanations are given, and other notations are adapted from the Results.

**S**_*N*_ gives the 2^*nd*^ Casimir of the Poincaré group, neither **S**_*P*_ nor **S**_*AP*_.

All three spins satisfy the *su*(2) algebra, i.e., $[{S}^{i},{S}^{j}]=i{\varepsilon }_{ijk}{S}^{k}$.

Since each suggested spin operator has its own strengths and weaknesses, Bauke *et al*.^27^ have suggested various electromagnetic environments to experimentally distinguish between the proposed relativistic spin operators. However, their experimental setup used electromagnetic interactions that would require a nontrivial change in the spin operators with momentum dependence by gauge coupling. On the other hand, a relativistic vortex can be generated from free electrons. The conflicting results of two recent works in refs. ^12,13^. mainly originating from using different relativistic operators motivate us to ask whether the problem of the proper spin and its corresponding OAM operators is related to the singular behaviour of relativistic vortex beams that can be determined experimentally.

In this study, we consider the two sets of operators, i.e., one set consisting of the new spin and OAM operators and another set consisting of the Dirac spin and OAM operators, because only two spin operators are derived from the parity-extended Poincaré group, i.e., the space-time symmetry^28^. For nonrelativistic electron vortices, we expect that the conserved OAM is essential to the existence of singular vortices. Because of Zitterbewegung^29^, expected for the Dirac position operator, the Dirac OAM is not a constant of motion. On the other hand, the particle position operator, which is obtained by a projection onto the particle subspace of the Dirac spinor space from the new position operator, shows no Zitterbewegung and, as a result, leads to conserved particle OAM. These different properties of the two kinds of position operators create different anticipations for the singularity of relativistic electron vortices and their experimental tests. The projected operators recently studied in ref. ^3^. will not be considered explicitly, because the projected operators will laed to the same results as the Dirac operators due to the fact that the expectation values of the projected operators are the same as those of the corresponding Dirac operators for electrons.

## Results

### Dirac spin and orbital angular momentum

In this section, for a clear comparison with the new spin and the corresponding OAM in the study of a relativistic vortex, we will briefly review the original Dirac theory^16^. As introduced by Dirac^16^, the total angular momentum is the sum of the Dirac spin

1 $${S}_{D}^{k}=\frac{{\Sigma }^{k}}{2}\equiv \left(\begin{array}{cc}\frac{{\sigma }^{k}}{2} & 0\\ 0 & \frac{{\sigma }^{k}}{2}\end{array}\right)$$

and the corresponding Dirac OAM, i.e., ${{\bf{r}}}_{D}\times {\bf{p}}$, where **r**_*D*_ is the Dirac position operator, which is the canonical operator represented by $i{\nabla }_{{\bf{p}}}$, and ${\sigma }^{k}$ ($k=\{x,y,z\}$) are the Pauli matrices. This total angular momentum is a constant of motion under the following Dirac Hamiltonian

2 $${H}_{D}=\alpha \cdot {\bf{p}}+\beta m,$$

where $\alpha \cdot {\bf{p}}={\alpha }^{j}{p}^{j}$. We use the Einstein summation convention in which we sum over repeated indices. For the Dirac Hamiltonian, the Dirac matrices are

3 $${\alpha }^{k}=(\begin{array}{cc}0 & {\sigma }^{k}\\ {\sigma }^{k} & 0\end{array}),\,\beta =(\begin{array}{cc}1 & 0\\ 0 & -\,1\end{array})$$

in the standard representation^16^. Here we use the natural unit $\hslash =c=1$. However, as noticed, the Dirac spin and the Dirac OAM are not separately conserved with the Dirac Hamiltonian, which is the reason why Dirac introduced spin angular momentum. That is,

4a $$[{H}_{D},{{\bf{S}}}_{D}]=i\alpha \times {\bf{p}},$$

4b $$[{H}_{D},{{\bf{r}}}_{D}\times {\bf{p}}]=-\,i\alpha \times {\bf{p}}.$$

The commutator of the Dirac OAM and the Dirac Hamiltonian in Eq. (4b) is not zero because the Dirac velocity operator $i[{H}_{D},{{\bf{r}}}_{D}]=\alpha$ is not proportional to the momentum **p**. This suggests that the existence of Zitterbewegung^29,30^, which is fast trembling motion first observed by Schrödinger, is closely related to the non-conservation of the Dirac OAM.

The Dirac Hamiltonian (2) gives the following well-known four solutions:

5 $$\begin{array}{ll}{u}^{1}({p}^{\mu })=\frac{1}{\sqrt{2m(E+m)}}(\begin{array}{c}E+m\\ 0\\ {p}^{z}\\ {p}^{x}+i{p}^{y}\end{array}), & {u}^{2}({p}^{\mu })=\frac{1}{\sqrt{2m(E+m)}}(\begin{array}{c}0\\ E+m\\ {p}^{x}-i{p}^{y}\\ -\,{p}^{z}\end{array}),\\ {u}^{3}({p}^{\mu })=\frac{1}{\sqrt{2m(E+m)}}(\begin{array}{c}-\,{p}^{z}\\ -\,{p}^{x}-i{p}^{y}\\ E+m\\ 0\end{array}), & {u}^{4}({p}^{\mu })=\frac{1}{\sqrt{2m(E+m)}}(\begin{array}{c}-\,{p}^{x}+i{p}^{y}\\ {p}^{z}\\ 0\\ E+m\end{array}),\end{array}$$

where ${p}^{\mu }=(E,{\bf{p}})$. ${u}^{1,2}({p}^{\mu })$ are the two positive energy spinors with energy eigenvalue $+E=\sqrt{{\bf{p}}\cdot {\bf{p}}+{m}^{2}}$, and ${u}^{3,4}({p}^{\mu })$ are the two negative energy spinors with energy eigenvalue $-E$. The eigenspinors satisfy the following orthogonality relation:

6 $${u}^{\gamma \dagger }({p}^{\mu }){u}^{\delta }({p}^{\mu })=\frac{E}{m}{\delta }_{\gamma \delta },$$

where *γ*, $\delta =\{1,2,3,4\}$, ${u}^{\gamma \dagger }({p}^{\mu })$ is the Hermitian conjugate of ${u}^{\gamma }({p}^{\mu })$, and ${\delta }_{\gamma \delta }$ is the Kronecker-delta function.

### The new spin and corresponding orbital angular momentum

Recently we derived the covariant spin operator of the parity-extended Poincaré group whose eigenstates provide the representation corresponding to a free massive elementary field with spin *s*^28^. The representation space of the parity-extended Poincaré group for free massive spin-1/2 fields is the four-spinor space in which the usual Dirac particle and antiparticle spinors reside^28,31^. In this section, we introduce the covariant spin operator as the new spin operator and the corresponding OAM in association with the new position operator.

The new spin operator was originally constructed by the generators of the Poincaré group; however to compare the differences between the new spin and the Dirac spin explicitly, it is convenient to represent the new spin operator in the Dirac four-spinor space by using the Dirac spin operator as^28^

7 $${S}_{N}^{k}=\frac{E}{m}{S}_{D}^{k}-\frac{{P}^{k}({{\bf{S}}}_{D}\cdot {\bf{P}})}{m(E+m)}+i{\gamma }^{5}\frac{1}{m}{({{\bf{S}}}_{D}\times {\bf{P}})}^{k},$$

where $${\gamma }^{5}=(\begin{array}{cc}0 & I\\ I & 0\end{array})$$ is the Dirac gamma matrix in the standard representation^31^, ${({{\bf{S}}}_{D}\times {\bf{P}})}^{k}$ is the *k*-component of the three-dimensional vector product ${{\bf{S}}}_{D}\times {\bf{P}}$ and *I* is the 2-dimensional identity matrix. We use upper case **P** for a momentum operator and lower case **p** for the eigenvalue of a momentum operator.

The following two fundamental dynamical equations were derived for a free massive spin-1/2 particle and antiparticle from the property of the parity operation^28^

8a $$({\gamma }^{\mu }{p}_{\mu }-m){\psi }_{P}({p}^{\mu })=0,$$

8b $$({\gamma }^{\mu }{p}_{\mu }+m){\psi }_{AP}({p}^{\mu })=0,\,{\rm{respectively}},$$

where ${\psi }_{P}({p}^{\mu })$ and ${\psi }_{AP}({p}^{\mu })$ are the particle and antiparticle spinors, respectively. These two Eqs. (8a) and (8b) are the same as the two covariant Dirac equations for particle and antiparticle spinors, where the Dirac gamma matrices are ${\gamma }^{0}=\beta$ and ${\gamma }^{k}=\beta {\alpha }^{k}$ in the standard representation^31^.

There are two positive energy and two negative energy solutions for each of Eqs. (8a) and (8b). Among these 8 solutions, the two particle eigenspinors are the same as ${u}^{1,2}({p}^{\mu })$ in Eq. (5), which are the positive-energy solutions of *H*_*D*_, and the two antiparticle eigenspinors are the following two negative energy solutions of Eq. (8b) ^31^:

9 $${v}^{1}({p}^{\mu })=\frac{1}{\sqrt{2m(E+m)}}(\begin{array}{c}{p}^{z}\\ {p}^{x}+i{p}^{y}\\ E+m\\ 0\end{array}),\,{v}^{2}({p}^{\mu })=\frac{1}{\sqrt{2m(E+m)}}(\begin{array}{c}{p}^{x}-i{p}^{y}\\ -\,{p}^{z}\\ 0\\ E+m\end{array}),$$

which are not the negative energy eigenstates ${u}^{3,4}({p}^{\mu })$ of *H*_*D*_. Then ${u}^{\mathrm{1,2}}({p}^{\mu })$ and ${v}^{\mathrm{1,2}}({p}^{\mu })$ satisfy the same orthogonality relation as ${u}^{j\dagger }({p}^{\mu }){u}^{k}({p}^{\mu })=E/m{\delta }_{jk}$ and ${v}^{j\dagger }({p}^{\mu }){v}^{k}({p}^{\mu })=E/m{\delta }_{jk}$. The four spinors ${u}^{1}({p}^{\mu })$, ${u}^{2}({p}^{\mu })$, ${v}^{1}({p}^{\mu })$, and ${v}^{2}({p}^{\mu })$ are Lorentz-boosted spinors from the rest spinors as

10 $$\begin{array}{ll}{u}^{1}({p}^{\mu })={e}^{{\gamma }^{5}\Sigma \cdot \zeta /2}(\begin{array}{c}1\\ 0\\ 0\\ 0\end{array}), & {u}^{2}({p}^{\mu })={e}^{{\gamma }^{5}\Sigma \cdot \zeta /2}(\begin{array}{c}0\\ 1\\ 0\\ 0\end{array}),\\ {v}^{1}({p}^{\mu })={e}^{{\gamma }^{5}\Sigma \cdot \zeta /2}(\begin{array}{c}0\\ 0\\ 1\\ 0\end{array}), & {v}^{2}({p}^{\mu })={e}^{{\gamma }^{5}\Sigma \cdot \zeta /2}(\begin{array}{c}0\\ 0\\ 0\\ 1\end{array}),\end{array}$$

where ${e}^{{\gamma }^{5}\Sigma \cdot \zeta /2}$ is a Lorentz boost with rapidity $\zeta =2\,\hat{{\bf{p}}}\,{\tanh }^{-1}\,[\sqrt{{\bf{p}}\cdot {\bf{p}}}/(E+m)]$. The new spin ${S}_{N}^{k}$ in Eq. (7) can also be expressed by using the following relation^28^

11 $${S}_{N}^{k}={e}^{{\gamma }^{5}\Sigma \cdot \zeta /2}\frac{{\Sigma }^{k}}{2}{e}^{-{\gamma }^{5}\Sigma \cdot \zeta /2}.$$

Then, it is easily seen that the four spinors ${u}^{\mathrm{1,2}}({p}^{\mu })$ and ${v}^{\mathrm{1,2}}({p}^{\mu })$ are also eigenstates of the new spin ${S}_{N}^{k}$ with the same eigenvalues of the rest spin Σ^*k*^/2 for the rest spinors ${u}^{\mathrm{1,2}}({k}^{\mu })$ and ${v}^{\mathrm{1,2}}({k}^{\mu })$, where ${k}^{\mu }=(m,{\bf{0}})$.

However, ${S}_{N}^{k}$ is not a good observable because it is not Hermitian, as seen from the last term in Eq. (7). This does not mean that ${S}_{N}^{k}$ is not a proper spin operator. In fact, ${S}_{N}^{k}$ becomes equivalent to the Hermitian particle spin operator ${S}_{P}^{k}$ and antiparticle spin operator ${S}_{AP}^{k}$ as they act on the particle states ${u}^{\mathrm{1,2}}({p}^{\mu })$ and the antiparticle states ${v}^{\mathrm{1,2}}({p}^{\mu })$, respectively^28^, i.e., (Methods 0.1.1)

12 $${S}_{N}^{k}{u}^{1,2}({p}^{\mu })={S}_{P}^{k}{u}^{1,2}({p}^{\mu })\,{\rm{and}}\,{S}_{N}^{k}{v}^{1,2}({p}^{\mu })={S}_{AP}^{k}{v}^{1,2}({p}^{\mu }),$$

with the explicit form of the particle and antiparticle spin operators

13 $$\begin{array}{rcl}{S}_{P}^{k} & = & {e}^{-{\gamma }^{0}{\gamma }^{5}\Sigma \cdot \zeta /2}\frac{{\Sigma }^{k}}{2}{e}^{{\gamma }^{0}{\gamma }^{5}\Sigma \cdot \zeta /2}={S}_{D}^{k}+\frac{{p}^{k}({\bf{p}}\cdot {{\bf{S}}}_{D})-{\bf{p}}\cdot {\bf{p}}{S}_{D}^{k}}{E(E+m)}+i\beta \frac{{({\bf{p}}\times \alpha )}^{k}}{E},\\ {S}_{AP}^{k} & = & {e}^{{\gamma }^{0}{\gamma }^{5}\Sigma \cdot \zeta /2}\frac{{\Sigma }^{k}}{2}{e}^{-{\gamma }^{0}{\gamma }^{5}\Sigma \cdot \zeta /2}={S}_{D}^{k}+\frac{{p}^{k}({\bf{p}}\cdot {{\bf{S}}}_{D})-{\bf{p}}\cdot {\bf{p}}{S}_{D}^{k}}{E(E+m)}-i\beta \frac{{({\bf{p}}\times \alpha )}^{k}}{E}\end{array}$$

in the momentum representation. Note that the particle and the antiparticle spin operators can be expressed by using the FW transformation matrix ${U}_{FW}({\bf{p}})$ because

14 $${e}^{{\gamma }^{0}{\gamma }^{5}\Sigma \cdot \zeta /2}=\frac{E+m+\beta \alpha \cdot {\bf{p}}}{\sqrt{2E(E+m)}}={U}_{FW}({\bf{p}}).$$

Thus, the particle spin ${S}_{P}^{k}$ is straightforwardly shown to be the same as the FW mean spin operator discussed in refs. ^13,17,23^.

It has been shown that ${S}_{N}^{k}$ gives Noether’s conserved spin angular momentum^28^. The conservation of spin can be also confirmed by using the commutators between spins and the corresponding Hamiltonians. Since the antiparticle spinors ${v}^{\mathrm{1,2}}({p}^{\mu })$ satisfy the different covariant Eq. (8b) from Eq. (8a) of ${u}^{\mathrm{1,2}}({p}^{\mu })$, the corresponding Hamiltonian for ${v}^{\mathrm{1,2}}({p}^{\mu })$ is also different from the original Dirac Hamiltonian, which is obtained as^31^

15 $${\tilde{H}}_{D}=-\,\alpha \cdot {\bf{p}}+\beta m.$$

Then one can easily check that the particle and antiparticle spins are conserved because the spins commute with the corresponding Hamiltonians as follows:

16 $$[{H}_{D},{S}_{P}^{k}]=0\,{\rm{and}}\,[{\tilde{H}}_{D},{S}_{AP}^{k}]=0.$$

This fact that the particle and antiparticle spins, ${S}_{P}^{k}$ and ${S}_{AP}^{k}$, are conserved quantities in each Hamiltonian implies that the corresponding conserved OAMs, which commute with the corresponding Hamiltonians ${H}_{D}$ and ${\tilde{H}}_{D}$, can be determined through the total angular momentum. On the other hand, one can see that the Dirac OAM does not commute with both ${H}_{D}$ and ${\tilde{H}}_{D}$.

To obtain the OAMs that commute with ${H}_{D}$ and ${\tilde{H}}_{D}$, it is necessary to define the new position operator **R**_*N*_ corresponding to the new spin operator **S**_*N*_ as^32^

17 $${{\bf{R}}}_{N}={e}^{{\gamma }^{5}\Sigma \cdot \zeta /2}{{\bf{r}}}_{D}{e}^{-{\gamma }^{5}\Sigma \cdot \zeta /2}.$$

**R**_*N*_ satisfies the same commutation relations as those of the Dirac position operator, i.e, $[{R}_{N}^{j},{R}_{N}^{k}]=0$ (locality condition), $[{R}_{N}^{j},{S}_{N}^{k}]=0$, and $[{R}_{N}^{j},{P}^{k}]=i{\delta }_{jk}$ for $j$, $k\in \{x,y,z\}$. The new position operator acting on the particle and the antiparticle states becomes the following Hermitian particle and antiparticle position operators, respectively,

18a $${R}_{P}^{k}={U}_{FW}^{\dagger }({\bf{p}}){r}_{D}^{k}{U}_{FW}({\bf{p}})={r}_{D}^{k}+\frac{i\beta {\alpha }^{k}}{2E}-\frac{i\beta {p}^{k}(\alpha \cdot {\bf{p}})+\sqrt{{\bf{p}}\cdot {\bf{p}}}{(\Sigma \times {\bf{p}})}^{k}}{2E(E+m)\sqrt{{\bf{p}}\cdot {\bf{p}}}}\,{\rm{and}}$$

18b $${R}_{AP}^{k}={U}_{FW}({\bf{p}}){r}_{D}^{k}{U}_{FW}^{\dagger }({\bf{p}})={r}_{D}^{k}+\frac{i\beta {\alpha }^{k}}{2E}-\frac{i\beta {p}^{k}(\alpha \cdot {\bf{p}})-\sqrt{{\bf{p}}\cdot {\bf{p}}}{(\Sigma \times {\bf{p}})}^{k}}{2E(E+m)\sqrt{{\bf{p}}\cdot {\bf{p}}}},$$

similar to the new particle and antiparticle spin operators ${S}_{P}^{k}$ and ${S}_{AP}^{k}$.

Subsequently, the velocity operators for the particle and the antiparticle are determined as

19a $$\frac{d{R}_{P}^{k}}{dt}=-\,i[{R}_{P}^{k},{H}_{D}]=\frac{{p}^{k}}{E}\frac{{H}_{D}}{E},$$

19b $$\frac{d{R}_{AP}^{k}}{dt}=-\,i[{R}_{AP}^{k},{\tilde{H}}_{D}]=\frac{{p}^{k}}{E}\frac{{\tilde{H}}_{D}}{E},$$

respectively. The particle and antiparticle spinors ${u}^{\mathrm{1,2}}({p}^{\mu })$ and ${v}^{\mathrm{1,2}}({p}^{\mu })$ are eigenstates of the above velocity operators with eigenvalues ${p}^{k}/E$ and −${p}^{k}/E$, respectively, because ${v}^{\mathrm{1,2}}({p}^{\mu })$ have negative energy eigenvalue −*E*. This shows that there is no Zitterbewegung for the new position operators. Consequently, the particle and antiparticle OAMs defined by ${{\bf{R}}}_{P/AP}\times {\bf{p}}$ are conserved by themselves. The OAM conservation of the free massive particles and antiparticles is also verified by the commutation relations:

20a $$[{L}_{P}^{k},{H}_{D}]={\varepsilon }_{klm}[{R}_{P}^{l}{P}^{m},{H}_{D}]=0,$$

20b $$[{L}_{AP}^{k},{\tilde{H}}_{D}]={\varepsilon }_{klm}[{R}_{AP}^{l}{P}^{m},{\tilde{H}}_{D}]=0,$$

where ${\varepsilon }_{klm}$ is the Levi-Civita symbol with ${\varepsilon }_{123}=1$.

### Existence of singular relativistic vortices and its implication

In the nonrelativistic case, free electron vortex states (with a phase singularity) carry a well-defined OAM, which requires the conservation of the OAM^1,2,33^. It is natural to expect that the conserved OAM is also essential for the existence of singular relativistic electron (Dirac particle) vortices. As studied in the previous sections, because of the Zitterbewegung of the Dirac position operator, the Dirac OAM is not conserved as shown in Eq. (4b). In constrast, the particle position operator shows no Zitterbewegung and, as a result, gives the conserved particle OAM in Eq. (20a). Therefore, the eigenstates of the particle OAM operator would compose the eigenstates of the particle Hamiltonian with a well-defined particle OAM such as those of the nonrelativistic case, but this is not the case for the eigenstates of the Dirac OAM. This raises the question: “could the existence of a singular relativistic vortex in an experiment be a probe for proper spin and position operators?” We call this question the’which operator question’. To answer the’which operator question’, a specific solution for relativistic beams is needed. Here, we focus on a Dirac particle (electron), because the reasoning for an antiparticle (positron) is parallel to that of a particle and straightforward.

Let us first consider the particle spin and the particle OAM, which admit the vortex solutions with well-defined OAM. We assume the relativistic beam to be paraxial, which propagates mainly along $z$-direction, i.e., $|{p}^{z}|\gg |{p}^{x}|,\,|{p}^{y}|$. The vortex solutions expressed in terms of the eigenstates of the particle OAM can be most easily studied in the FW representation for electrons, because the particle position and the particle OAM operators are represented in the usual canonical form in the FW representation as

21a $${r}^{k}={U}_{FW}({\bf{P}}){R}_{P}^{k}{U}_{FW}^{\dagger }({\bf{P}})=i{\partial }_{{p}^{k}},$$

21b $${L}^{z}={U}_{FW}({\bf{P}}){L}_{P}^{z}{U}_{FW}^{\dagger }({\bf{P}})=-\,i{\partial }_{\phi },$$

from Eq. (18a), where ${\bf{P}}=-\,i\nabla$, ${\partial }_{{p}^{k}}=\partial /(\partial {p}^{k})$, ${\partial }_{\phi }=\partial /(\partial \phi )$, and $\phi$ is the azimuthal angle of the cylindrical coordinate $(\rho ,\phi ,z)$ in the FW representation.

The FW transformations of the state $\psi (x)$ and the Dirac Hamiltonian *H*_*D*_ in the original representation are performed as

22a $${\psi }_{FW}(x)=\sqrt{\frac{m}{E}}{U}_{FW}(\,-\,i\nabla )\psi (x),$$

22b $${H}_{FW}={U}_{FW}(\,-\,i\nabla ){H}_{D}{U}_{FW}^{\dagger }(\,-\,i\nabla )=\beta E,$$

where $\psi (x)$ is the eigenstate of the Dirac Hamiltonian ${H}_{D}$ with eigenvalue $E$ and $x=(t,{\bf{x}})$, with $t$ being time. The FW transformation of the state in Eq. (22a) differs from the FW transformation of the state in other studies^13,17,23^ by the normalization factor $\sqrt{m/E}$, which reflects that ${u}^{\dagger j}({p}^{\mu }){u}^{k}({p}^{\mu })=(E/m){\delta }_{jk}$ of the eigenspinors of the Dirac Hamiltonian ${H}_{D}$ is frame-dependent but ${u}_{FW}^{\dagger j}{u}_{FW}^{k}={\delta }_{jk}$ of the eigenspinors of the Hamiltonian ${H}_{FW}$ in the FW representation is frame-independent because the eigenspinors ${u}_{FW}^{1}={(1,0,0,0)}^{T}$ and ${u}_{FW}^{2}={(0,1,0,0)}^{T}$ do not depend on the reference frame, where T is the transpose of the matrix. The eigenspinors ${u}_{FW}^{1}$ and ${u}_{FW}^{2}$ are also eigenspinors of the spin Σ/2 in the FW representation, because Σ/2 commutes with ${H}_{FW}$.

In the FW representation the solutions for the Dirac equation in Eq. (8a) with the Hamiltonian ${H}_{FW}$ in Eq. (22b) have the following form in the cylindrical coordinate $(\rho ,\phi ,z)$:

23 $${\psi }_{FW}(x)={e}^{-iEt}{\psi }_{FW}(\rho ,z){e}^{il\phi }(a{u}_{FW}^{1}+b{u}_{FW}^{2}),$$

where $|a{|}^{2}+|b{|}^{2}=1$. We consider the solution ${\psi }_{FW}(x)$ to be monoenergetic with energy $E$ for simplicity. Then, it is sufficient to analyse only the spatial dependence of ${\psi }_{FW}(x)$, i.e.,

24 $${\psi }_{FW}({\bf{x}})={\psi }_{FW}(\rho ,z){e}^{il\phi }(a{u}_{FW}^{1}+b{u}_{FW}^{2}),$$

where ${\bf{x}}=(\rho ,\phi ,z)$. Note that Bialynicki-Birula *et al*.^12^ showed that $[{L}_{D}^{z},{H}_{D}]\psi =0$ leads to no vortex solution for relativistic electrons, because ${[{L}_{D}^{z},{H}_{D}]}^{2}=0$ becomes ${({p}^{x})}^{2}+{({p}^{y})}^{2}=0$, which provides the Laplace equation and not the paraxial equation. Here ${L}_{D}^{z}$ is the $z$-component of the Dirac OAM, ${{\bf{r}}}_{D}\times {\bf{p}}$. However, the null commutator in Eq. (20a) for the *z*-component of the particle OAM does not derive ${({p}^{x})}^{2}+{({p}^{y})}^{2}=0$, because $[{R}_{P}^{i},{\alpha }^{j}]\ne 0$ as can be seen from Eq. (18a) unlike $[{r}_{D}^{i},{\alpha }^{j}]=0$ of the Dirac position operator for *i*, $j\in \{x,y,z\}$.

One should be careful to calculate the expectation values of the operator in the FW representation to maintain the physical equivalence between the original representation and FW representation for superposition states with different momenta, because ${U}_{FW}^{\dagger }({\bf{p}}{\prime} ){U}_{FW}({\bf{p}})\ne 1$ for ${\bf{p}}{\prime} \ne {\bf{p}}$ even though ${U}_{FW}^{\dagger }({\bf{p}}){U}_{FW}({\bf{p}})=1$. The expectation value of the operator ${\mathscr{O}}$ at **x** in the original representation given by ${\langle {\mathscr{O}}\rangle }_{{\bf{x}}}\equiv {\psi }^{\dagger }({\bf{x}}){\mathscr{O}}\psi ({\bf{x}})$ is not simply the same as ${\psi }_{FW}^{\dagger }({\bf{x}}){{\mathscr{O}}}_{FW}{\psi }_{FW}({\bf{x}}),$ where ${{\mathscr{O}}}_{FW}={U}_{FW}(\,-\,i\nabla ){\mathscr{O}}{U}_{FW}^{\dagger }(\,-\,i\nabla )$ is the operator representative in the FW representation. The right expression in the FW representation is (Methods 0.1.2)

25 $${\langle {\mathscr{O}}\rangle }_{{\bf{x}}}={\psi }_{FW}^{\dagger }({\bf{x}})\frac{E}{m}{U}_{FW}(i\overleftarrow{\nabla }){U}_{FW}^{\dagger }(\,-\,i\nabla ){{\mathscr{O}}}_{FW}{\psi }_{FW}({\bf{x}}),$$

where $\overleftarrow{\nabla }$ operates on ${\psi }_{FW}^{\dagger }({\bf{x}})$ to the left. This relation provides a nontrivial spin-orbit interaction effect in terms of the Dirac spin and the Dirac OAM rather than the new (particle) spin and the new (particle) OAM. The normalized expectation value of ${\mathscr{O}}$ is divided by the probability amplitude ${\psi }^{\dagger }({\bf{x}})\psi ({\bf{x}})$, which is also not the same as ${\psi }_{FW}^{\dagger }({\bf{x}}){\psi }_{FW}({\bf{x}})$ in general (Methods 0.1.2).

The singularity of the vortex is encoded in the rotational property of the velocity around the propagating direction. The expectation value of the particle velocity operator in Eq. (19a) at **x**, which we call the particle velocity at **x**, is written as

26 $${\langle {\bf{v}}\rangle }_{{\bf{x}}}={\rm{Re}}\left\{\frac{{\psi }^{\dagger }({\bf{x}})\frac{{\bf{P}}}{E}\psi ({\bf{x}})}{{\psi }^{\dagger }({\bf{x}})\psi ({\bf{x}})}\right\}.$$

For a furthur calculation, we use the paraxial approximation of the Hamiltonian ${H}_{FW}$ in ref. ^13^. with $E=\sqrt{{p}_{0}^{2}+{m}^{2}}$, i.e.,

27 $${H}_{FW}=\sqrt{{\bf{p}}\cdot {\bf{p}}+{m}^{2}}\approx \sqrt{{p}_{0}^{2}+{m}^{2}}+\frac{{({p}^{x})}^{2}+{({p}^{y})}^{2}}{2\sqrt{{p}_{0}^{2}+{m}^{2}}}+\frac{{p}_{0}({p}^{z}-{p}_{0})}{\sqrt{{p}_{0}^{2}+{m}^{2}}},$$

where ${p}_{0}\approx {p}^{z}$; then, we obtain the following local Laguerre-Gauss (LG) solution with the form

28 $$\begin{array}{rcl}{\psi }_{FW}({\bf{x}}) & = & {e}^{i{p}_{0}z}{\tilde{\psi }}_{FW}({\bf{x}})\\  & = & {e}^{i{p}_{0}z}\frac{1}{{w}^{|l|+1}(z)}\,\exp \,\left[\,-\,\frac{{\rho }^{2}}{{w}^{2}(z)}\right]{L}_{n}^{|l|}\left(\frac{2{\rho }^{2}}{{w}^{2}(z)}\right)\\  &  & \times \,{\rho }^{|l|}{e}^{il\phi }\,\exp \,[-i(2n+|l|+1)\,{\tan }^{-1}\,\left(\frac{z}{{z}_{0}}\right)\\  &  & +\,i\frac{2z{\rho }^{2}}{{z}_{0}w(z)}](a{u}_{FW}^{1}+b{u}_{FW}^{2}),\end{array}$$

where $w(z)$ is the beam width, ${z}_{0}=({p}_{0}w{(0)}^{2})/2$, and ${\tilde{\psi }}_{FW}({\bf{x}})$ satisfies the paraxial wave equation^1,13^. Bliokh *et al*.^18^ criticized the use of Barnett’s LG solution, because Barnett did not correctly describe the *z*-component of the magnetic moment of the electron. However, this is not due to the use of the LG solution but rather due to the incorrect calculation of the expectation value of ${({{\bf{r}}}_{D}\times {\bf{p}})}^{z}$. The correct calculation of this operator is shown in the Methods 0.3, in which the spin-orbit interaction (SOI) effects are clearly presented in operator form. The expectation values of ${p}^{x,y}$ at **x** are nonzero for the LG solution, even though the expectation values of ${p}^{x,y}$ over all space are zero, which shows the SOI effects. This implies that the LG solution could be a suitable expression of an experimental relativistic paraxial electron beam.

In the paraxial approximation, the state ${\psi }_{FW}({\bf{x}})$ in Eq. (28) varies only gradually along the $z$-axis such that

29 $${\partial }_{z}{\psi }_{FW}({\bf{x}})={p}_{0}{\psi }_{FW}({\bf{x}})+{e}^{i{p}_{0}z}{\partial }_{z}{\tilde{\psi }}_{FW}({\bf{x}})\approx {p}_{0}{\psi }_{FW}({\bf{x}}).$$

That is, the $z$ dependence of the solution can be considered solely by ${e}^{i{p}_{0}z}$ and $w(z)$ can be replaced by $w(0)\equiv w$. We are interested in the singular behaviour of the relativistic wave solutions for $\rho \to 0$, hence the region of $\rho  < w/\sqrt{2}$ will be considered. On the other hand, $\rho$ should be greater than 1/*m*, the Compton wavelength, because one particle theory is not valid in the region less than the Compton wavelength in which pair production is inevitable. Thus, in our study, we refer to the region of the vortex solution determined by

30 $$\frac{1}{m} < \rho  < \frac{w}{\sqrt{2}}$$

as the physical vortex region for simplicity. In the physical vortex region, the wavefunction ${\psi }_{FW}({\bf{x}})$ in Eq. (28) can be written as

31 $${\psi }_{FW}({\bf{x}})\approx {d}_{0}(l){e}^{i{p}_{0}z}\frac{1}{{w}^{|l|+1}}{\rho }^{|l|}{e}^{il\phi }(a{u}_{FW}^{1}+b{u}_{FW}^{2})$$

for the associated Laguerre polynomial

32 $${L}_{n}^{|l|}\left(\frac{2{\rho }^{2}}{{w}^{2}}\right)={d}_{0}(l)+\cdots {d}_{n}{\left(\frac{2{\rho }^{2}}{{w}^{2}}\right)}^{n},$$

where ${d}_{0}(l)$ is a function of *l* and *d*_*n*_ is constant.

The physical velocity is actually defined in the original representation; thus, we should use $\psi ({\bf{x}})$ that is obtained from the inverse of the FW transformation in Eq. (22a). In the physical vortex region, the denominator of the particle velocity in Eq. (26) can be simplified as (Methods 0.1.2)

33 $${\psi }^{\dagger }({\bf{x}})\psi ({\bf{x}})\approx \frac{E}{m}{\psi }_{FW}^{\dagger }({\bf{x}}){\psi }_{FW}({\bf{x}})\approx \frac{E}{m}{\left[{d}_{0}(l)\frac{{\rho }^{|l|}}{{w}^{|l|+1}}\right]}^{2}.$$

Additionally, the numerator of the particle velocity $({v}^{x},{v}^{y},{v}^{z})$ at **x** in Eq. (26) can be obtained as (Methods 0.2)

34 $${\psi }^{\dagger }({\bf{x}})\left(\frac{{p}^{x}}{E},\frac{{p}^{y}}{E},\frac{{p}^{z}}{E}\right)\psi ({\bf{x}})\approx {\left[{d}_{0}(l)\frac{{\rho }^{|l|}}{{w}^{|l|+1}}\right]}^{2}\left(\,-\,\frac{ly}{m{\rho }^{2}},\frac{lx}{m{\rho }^{2}},\frac{{p}_{0}}{m}\right).$$

As a result, the particle velocity at **x** is given as

35 $${\langle {\bf{v}}\rangle }_{{\bf{x}}}=\left(\,-\,\frac{ly}{E{\rho }^{2}},\frac{lx}{E{\rho }^{2}},\frac{{p}_{0}}{E}\right).$$

This particle velocity at **x** describes that electrons move along the *z*-direction with spiral circular motion, which represents the singular vortex motion along the *z*-axis. This result shows that the relativistic vortex solution interpreted by the particle velocity supports the singular vortex like the nonrelativistic vortex with the following circulation^14,15^

36 $${\Gamma }_{P}={\oint }_{C}\,{\langle {\bf{v}}\rangle }_{{\bf{x}}}\cdot d{\bf{l}}=2\pi \frac{l}{E},$$

where *C* is an arbitrary closed path around the *z*-axis.

Next, we study the singularity of the vortex solutions in Eq. (28) by using the Dirac position and the Dirac velocity. The Dirac velocity at **x** is obtained as (Methods 0.2)

37 $$\begin{array}{rcl}{\langle {{\bf{v}}}_{D}\rangle }_{{\bf{x}}} & = & \frac{{\psi }^{\dagger }({\bf{x}})\alpha \psi ({\bf{x}})}{{\psi }^{\dagger }({\bf{x}})\psi ({\bf{x}})}\\  & = & \frac{1}{2E{\psi }_{FW}^{\dagger }({\bf{x}}){\psi }_{FW}({\bf{x}})}[(i\nabla {\psi }_{FW}^{\dagger }({\bf{x}})){\psi }_{FW}({\bf{x}})-{\psi }_{FW}^{\dagger }({\bf{x}})(i\nabla {\psi }_{FW}({\bf{x}}))\\  &  & +2\nabla \times ({\psi }_{FW}^{\dagger }({\bf{x}}){S}_{D}^{k}{\psi }_{FW}({\bf{x}}))]\\  & \approx  & \frac{1}{E}\left(\,-\,\frac{ly}{{\rho }^{2}}(1\mp 2\langle {S}_{D}^{z}\rangle ),\frac{lx}{{\rho }^{2}}(1\mp 2\langle {S}_{D}^{z}\rangle ),{p}_{0}\right),\end{array}$$

where $\mp$ corresponds to positive and negative $l$, respectively. Here the part proportional to the expectation value of the $z$-component of the Dirac spin operator $\langle {S}_{D}^{z}\rangle$ is related to the Zitterbewegung. Although the Zitterbewegung in the Dirac position operator might appear only within the Compton wavelength, the result of this strange fast microscopic motion can survive as an observable spin effect. This was the original notion of Zitterbewegung proposed by Schrödinger, i.e., spin may be considered as orbital angular momentum of the electron performing Zitterbewegung, because ${{\bf{S}}}_{D}=(\,-\,i/4)\alpha \times \alpha$^29,34^. $\langle {S}_{D}^{z}\rangle =({a}^{\ast }{u}_{FW}^{1\dagger }+{b}^{\ast }{u}_{FW}^{2\dagger }){S}_{D}^{z}(a{u}_{FW}^{1}+b{u}_{FW}^{2})$ becomes approximately equal to $\frac{{\psi }^{\dagger }(x){S}_{D}^{z}\psi (x)}{{\psi }^{\dagger }(x)\psi (x)}$ for the paraxial condition $|{p}^{z}|\gg |{p}^{x}|,|{p}^{y}|$, which can also be expected from the eigenspinors in Eq. (5). Similar to the particle velocity, the Dirac velocity at **x** indicates that electrons move along the *z*-direction with a spiral motion. However, in contrast to the particle velocity, the spiral motion described by the $x$- and $y$-components of the Dirac velocity depends on the expectation value of the $z$-component of the Dirac spin, i.e., the Dirac spin orientation. The circulation for the Dirac velocity

38 $${\Gamma }_{D}={\oint }_{C}\,{\langle {{\bf{v}}}_{D}\rangle }_{{\bf{x}}}\cdot d{\bf{l}}=2\pi \frac{l}{E}(1\mp 2\langle {S}_{D}^{z}\rangle )$$

shows that the Dirac spin orientation determines whether a singular vortex exists.

**Figure 1:**
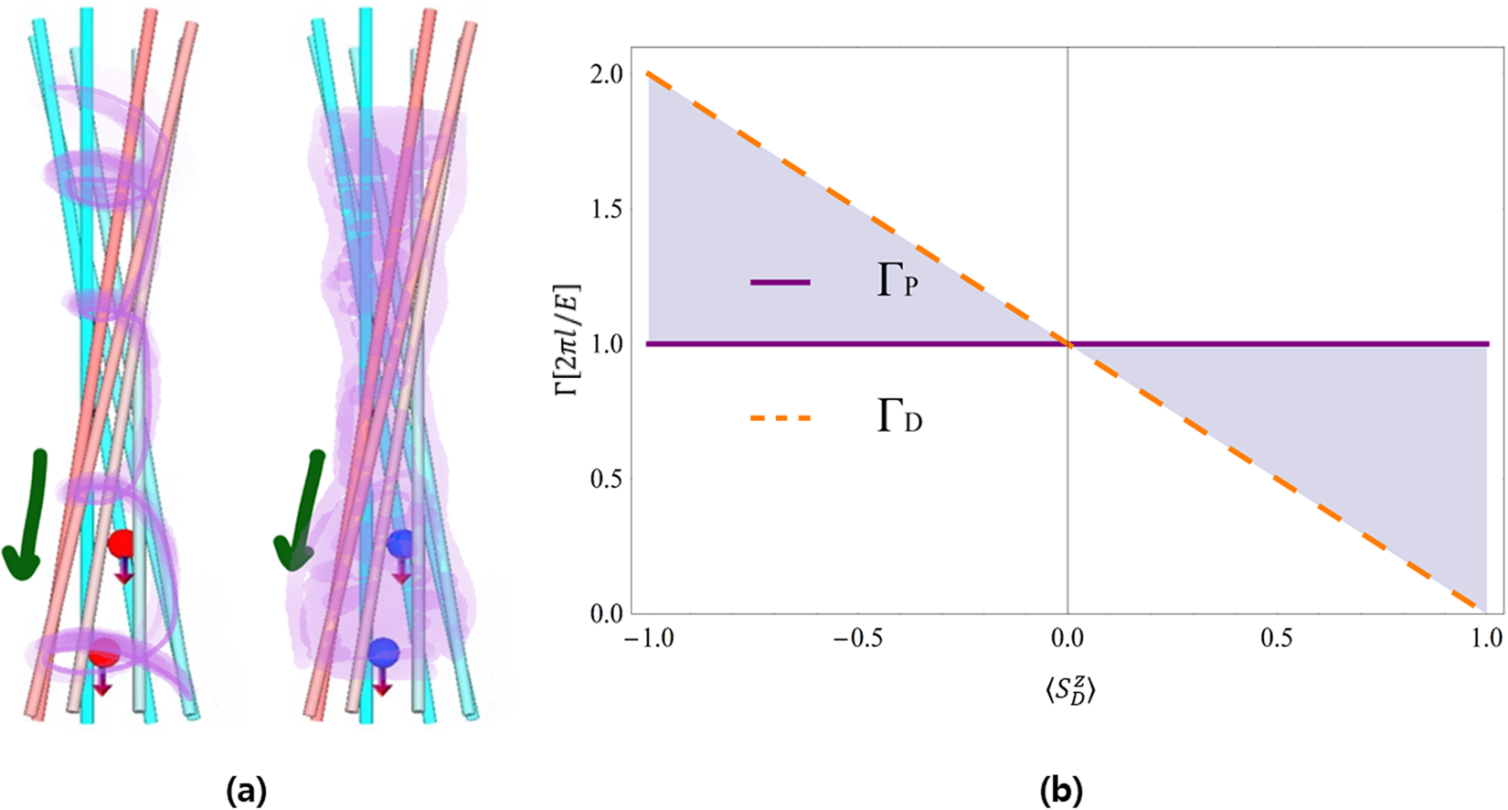
(**a**) Schematic diagrams of spin-polarized paraxial beams for the particle velocity (left) and the Dirac velocity (right). (**b**) Spin-orientation dependence of the circulations of the particle velocity Γ_*p*_ and the Dirac velocity Γ_*D*_. This clearly shows the independence on the Dirac spin orientation for the particle velocity and the strong dependence on the Dirac spin orientation for the Dirac velocity.

For a comparison with the circulation of the particle velocity Γ_*P*_, we plot the circulation of the Dirac velocity Γ_*D*_ as a function of twice the Dirac spin orientation $2\langle {S}_{D}^{z}\rangle$ for positive $l$ in Fig. 1(b). Figure 1(b) clearly shows that Γ_*P*_ does not depend on the Dirac spin orientation, but Γ_*D*_ depends on the Dirac spin orientation. If the Dirac spin is unpolarized or the Dirac spin orientation is in the $xy$-plane perpendicular to the propagating direction of the electron beam, Γ_*D*_ is the same as Γ_*P*_. However, compared with Γ_*P*_, Γ_*D*_ can be stronger if the angle between the Dirac spin orientation and the propagating direction of the electron beam is obtuse or weaker if acute. In particular, if the Dirac spin orientation is parallel to the propagating direction of the electron beam, the spiral circular motion of the electron beam disappears, i.e., Γ_*D*_ = 0 (Fig. 1(b)).

The Dirac velocity may be distinguished from the particle velocity experimentally by the characteristic spin orientation-dependent property. In particular, in order to answer the’which operator question’, for instance, two different setups can be considered in using spin-polarized electron beams moving along the $z$-direction in relativistic vortex experiments. In one setup, the spin is antiparallel to the propagating direction, and in the other setup, the spin is parallel to the propagating direction. As discussed in Fig. 1(b), for the particle velocity, the two setups will give the same vortex structure independent of the spin. However, for the Dirac velocity, the two setups will give very different observations of electron beams; i.e., the antiparallel spin gives spiral circular currents leading a vortex structure, but the parallel spin gives non-spiral circular currents resulting in no vortex structure. The probability current given by the particle velocity in Eq. (35) forms a whirlpool around the singularity, but the current given by the Dirac velocity in Eq. (37) with spin parallel to the propagating direction does not form a whirlpool. The probability density in Eq. (33) shows typical behaviour near the phase singularity^1^. The magnetic field induced by the whirlpool motion can be detected in an experiment. Thus, whether the non-vortex structure exists can play the role of a smoking-gun in distinguishing which ones can be proper relativistic operators. Consequently, distinguishable experimental observation results of a relativistic vortex in such two different setups could provide a clear answer to the question regarding proper relativistic observables, i.e., position, spin, and OAM. In addition, such an experimental answer to the long-standing question regarding proper relativistic observables could also provide reliable evidence to clarify whether Zitterbewegung is a real physical effect. Similar results are expected in relativistic proton and positron vortices for the new operators using parallel logic.

## Conclusion

We have studied the singularity of relativistic electron vortex beams using two different sets of relativistic operators. The first set includes the particle position, spin, and OAM operators that admit well-defined OAM $l$ for an LG vortex solution in the FW representation. The particle operators predict a singularity in the circulation of the inverse FW transformed relativistic LG vortex solution to the original representation, which shows the same feature of the Schrödinger nonrelativistic vortex. The second set consists of the usual Dirac position, spin, and OAM operators by which the spin orientation-dependent singularity of the same vortex solution is anticipated.

It was predicted that spin seems to have little effect in the study of a relativistic electron vortex beam for typical parameters in state-of-the art transmission electron microscopy experiments based on the estimation of the particle density ${\psi }^{\dagger }({\bf{x}})\psi ({\bf{x}})$ in the paraxial regime^35^. Actually, our study shows a similar result where ${\psi }^{\dagger }({\bf{x}})\psi ({\bf{x}})$ has a considerable spin effect in Eq. (47) (Methods 0.1.2) if $\rho$ is smaller than the Compton wavelength, i.e., $\rho  < 1/m$, while the spin effect can be negligible in ${\psi }^{\dagger }({\bf{x}})\psi ({\bf{x}})$ in Eq. (33) for the physical region, i.e., $1/m < \rho  < w/\sqrt{2}$, of interest to us. However, in sharp contrast to the behaviour of the particle density ${\psi }^{\dagger }({\bf{x}})\psi ({\bf{x}})$ in the paraxial regime, as discussed in the Results, the behaviours of the particle velocity and the Dirac velocity exhibit crucial differences due to the spin. Then, we discussed a possible experimental setup to probe a proper set of relativistic observables based on the very different predictions from the two sets of relativistic operators for the singularity of the LG vortex solution. Especially for a paraxial electron beam with spin parallel to the propagating direction, it could be experimentally distinguished that for the Dirac operators, singularity- and vortex-like motion do not exist, but for the particle operators, a singular vortex exists. Therefore, such spin-polarized relativistic electron vortex beam experiments could provide an answer to the question: which relativistic observables are proper?

## Methods

### Equivalent expressions between the original and the FW representation

#### New spin, particle spin and antiparticle spin

Let ${u}^{1}({k}^{\mu })={(1,0,0,0)}^{T}$, ${u}^{2}({k}^{\mu })={(0,1,0,0)}^{T}$, ${v}^{1}({k}^{\mu })={(0,0,1,0)}^{T}$, and ${v}^{2}({k}^{\mu })={(0,0,0,1)}^{T}$. Then the operators $(1\pm {\gamma }^{0})/2$ project the rest spinors onto particle and antiparticle subspace, respectively, i.e.,

39a $$\frac{1+{\gamma }^{0}}{2}{u}^{1,2}({k}^{\mu })={u}^{1,2}({k}^{\mu }),\,\frac{1+{\gamma }^{0}}{2}{v}^{1,2}({k}^{\mu })=0$$

39b $$\frac{1-{\gamma }^{0}}{2}{v}^{1,2}({k}^{\mu })={v}^{1,2}({k}^{\mu }),\,\frac{1-{\gamma }^{0}}{2}{u}^{1,2}({k}^{\mu })=0.$$

By using the relation

40 $${e}^{{\gamma }^{5}{\boldsymbol{\Sigma }}\cdot \zeta \mathrm{/2}}\frac{1\pm {\gamma }^{0}}{2}=\sqrt{\frac{E}{m}}{e}^{\mp {\gamma }^{0}{\gamma }^{5}{\boldsymbol{\Sigma }}\cdot \zeta \mathrm{/2}}\frac{1\pm {\gamma }^{0}}{2},$$

and from Eqs. (10) and (11), one can obtain

41a $$\begin{array}{rcl}{S}_{N}^{k}{u}^{1,2}({p}^{\mu }) & = & {e}^{{\gamma }^{5}\Sigma \cdot \zeta /2}\frac{{\Sigma }^{k}}{2}\frac{1+{\gamma }^{0}}{2}{u}^{1,2}({k}^{\mu })\\  & = & \sqrt{\frac{E}{m}}{e}^{-{\gamma }^{0}{\gamma }^{5}\Sigma \cdot \zeta /2}\frac{{\Sigma }^{k}}{2}\frac{1+{\gamma }^{0}}{2}{u}^{1,2}({k}^{\mu })\\  & = & {e}^{-{\gamma }^{0}{\gamma }^{5}\Sigma \cdot \zeta /2}\frac{{\Sigma }^{k}}{2}{e}^{{\gamma }^{0}{\gamma }^{5}\Sigma \cdot \zeta /2}{u}^{1,2}({p}^{\mu })\\  & = & {S}_{P}^{k}{u}^{1,2}({p}^{\mu }),\end{array}$$

41b $$\begin{array}{rcl}{S}_{N}^{k}{v}^{1,2}({p}^{\mu }) & = & {e}^{{\gamma }^{5}\Sigma \cdot \zeta /2}\frac{{\Sigma }^{k}}{2}\frac{1-{\gamma }^{0}}{2}{v}^{1,2}({k}^{\mu })\\  & = & \sqrt{\frac{E}{m}}{e}^{{\gamma }^{0}{\gamma }^{5}\Sigma \cdot \zeta /2}\frac{{\Sigma }^{k}}{2}\frac{1-{\gamma }^{0}}{2}{v}^{1,2}({k}^{\mu })\\  & = & {e}^{{\gamma }^{0}{\gamma }^{5}\Sigma \cdot \zeta /2}\frac{{\Sigma }^{k}}{2}{e}^{-{\gamma }^{0}{\gamma }^{5}\Sigma \cdot \zeta /2}{v}^{1,2}({p}^{\mu })\\  & = & {S}_{AP}^{k}{v}^{1,2}({p}^{\mu }).\end{array}$$

The first line of the above equation corresponds to the same relation in ref. ^23^.

#### Useful expressions

Let us consider the Fourier transformation in the original representation

42 $$\psi ({\bf{x}})=\int \,{d}^{3}p{e}^{i{\bf{p}}\cdot {\bf{x}}}\psi ({\bf{p}}).$$

Then the state in the FW representation becomes

43 $${\psi }_{FW}({\bf{x}})=\sqrt{\frac{m}{E}}{U}_{FW}(\,-\,i\nabla )\psi ({\bf{x}})=\int \,{d}^{3}p\sqrt{\frac{m}{E}}{e}^{i{\bf{p}}\cdot {\bf{x}}}{U}_{FW}({\bf{p}})\psi ({\bf{p}}).$$

Hence the expectation value of the operator ${\mathscr{O}}$ at **x** in the original representation becomes

44 $$\begin{array}{rcl}{\langle {\mathscr{O}}\rangle }_{x} & = & \int \,{d}^{3}p{d}^{3}p{\prime} {e}^{-i{\bf{p}}{\prime} \cdot {\bf{x}}}{\psi }^{\dagger }({\bf{p}}{\prime} ){\mathscr{O}}{e}^{i{\bf{p}}\cdot {\bf{x}}}\psi ({\bf{p}})\\  & = & \frac{E}{m}\,\int \,{d}^{3}p{d}^{3}p{\prime} {e}^{-i{\bf{p}}{\prime} \cdot {\bf{x}}}{e}^{i{\bf{p}}\cdot {\bf{x}}}{\psi }_{FW}^{\dagger }({\bf{p}}{\prime} ){U}_{FW}({\bf{p}}{\prime} ){\mathscr{O}}{U}_{FW}^{\dagger }({\bf{p}}){\psi }_{FW}({\bf{p}})\\  & = & \frac{E}{m}{\psi }_{FW}^{\dagger }({\bf{x}}){U}_{FW}(i\overleftarrow{\nabla }){\mathscr{O}}{U}_{FW}^{\dagger }(\,-\,i\nabla ){\psi }_{FW}({\bf{x}})\\  & = & \frac{E}{m}{\psi }_{FW}^{\dagger }({\bf{x}}){U}_{FW}(i\overleftarrow{\nabla }){U}_{FW}^{\dagger }(\,-\,i\nabla ){{\mathscr{O}}}_{FW}{\psi }_{FW}({\bf{x}})\end{array}$$

for $${\mathscr{O}}={U}_{FW}^{\dagger }(\,-\,i\nabla ){{\mathscr{O}}}_{FW}{U}_{FW}(\,-\,i\nabla )$$.

Next let us calculate the probability density at **x**:

45 $$\begin{array}{l}{\psi }^{\dagger }({\bf{x}})\psi ({\bf{x}})\\ \begin{array}{rcl} & = & \int \,{d}^{3}p{d}^{3}p{\prime} {e}^{-i{\bf{p}}{\prime} \cdot {\bf{x}}}{e}^{i{\bf{p}}\cdot {\bf{x}}}{\psi }_{FW}^{\dagger }({\bf{p}}{\prime} )\frac{E}{m}{U}_{FW}({\bf{p}}{\prime} ){U}_{FW}^{\dagger }({\bf{p}}){\psi }_{FW}({\bf{p}})\\  & = & \frac{E+m}{2m}{\psi }_{FW}^{\dagger }({\bf{x}}){\psi }_{FW}({\bf{x}})+\frac{1}{2m(E+m)}[(\nabla {\psi }_{FW}^{\dagger }({\bf{x}}))\cdot (\nabla {\psi }_{FW}({\bf{x}}))\\  &  & -\,i(\nabla {\psi }_{FW}^{\dagger }({\bf{x}}))\cdot \Sigma \times (\nabla {\psi }_{FW}({\bf{x}}))]\end{array}\end{array}$$

using

46 $${U}_{FW}({\bf{p}})=\frac{E+m+\beta \alpha \cdot {\bf{p}}}{\sqrt{2E(E+m)}},\,{U}_{FW}({\bf{p}}{\prime} ){U}_{FW}^{\dagger }({\bf{p}})\to \frac{{(E+m)}^{2}-\beta \alpha \cdot {\bf{p}}{\prime} \beta \alpha \cdot {\bf{p}}}{2E(E+m)},$$

where we have used the fact that the expectation values of the odd terms, which have no diagonal elements, for ${\psi }_{FW}({\bf{p}})$ become zero. The ${\psi }^{\dagger }({\bf{x}})\psi ({\bf{x}})$ for the LG solution in Eq. (28) approximately becomes

47 $$\begin{array}{rcl}{\psi }^{\dagger }({\bf{x}})\psi ({\bf{x}}) & \approx  & \frac{E}{m}{\psi }_{FW}^{\dagger }({\bf{x}}){\psi }_{FW}({\bf{x}})\\  &  & -\,i\frac{1}{2m(E+m)}(\nabla {\psi }_{FW}^{\dagger }({\bf{x}}))\cdot \Sigma \times (\nabla {\psi }_{FW}({\bf{x}}))\\  & \approx  & \frac{E}{m}\frac{{\rho }^{|l|}}{{w}^{2(|l|+1)}}{d}_{0}{(l)}^{2}\frac{{\rho }^{|l|}}{{w}^{2(|l|+1)}}\\  &  & +\,\frac{1}{m(E+m)}{p}^{0}l\langle {\Sigma }^{\phi }\rangle \frac{{\rho }^{|l|}}{{w}^{2(|l|+1)}}{d}_{0}{(l)}^{2}\frac{{\rho }^{|l|-1}}{{w}^{2(|l|+1)}}\end{array}$$

for Σ^*ϕ*^ = −sin *ϕ* Σ^*x*^ + cos *ϕ* Σ^*y*^ with the paraxial condition in the physical region. The second term in the last line becomes greater than the first when the $\rho$ satisfies $\rho  < l/(E+m) < 1/m$, i.e., less than the Compton wavelength. Therefore, the final expression in the physical vortex region becomes

48 $${\psi }^{\dagger }({\bf{x}})\psi ({\bf{x}})\approx \frac{E}{m}\frac{{\rho }^{|l|}}{{w}^{2(|l|+1)}}{d}_{0}{(l)}^{2}\frac{{\rho }^{|l|}}{{w}^{2(|l|+1)}}\approx \frac{E}{m}{\psi }_{FW}^{\dagger }({\bf{x}}){\psi }_{FW}({\bf{x}}).$$

### The particle velocity and the Dirac velocity

Let us calculate the numerator of the particle velocity operator:

49 $$\begin{array}{rcl}{\rm{Re}}\,[{\psi }^{\dagger }({\bf{x}})\frac{p}{E}\psi ({\bf{x}})] & = & -\,\frac{i}{2E}[{\psi }^{\dagger }({\bf{x}})\nabla \psi ({\bf{x}})-(\nabla {\psi }^{\dagger }({\bf{x}}))\psi ({\bf{x}})]\\  & = & \frac{1}{2m}\,\int \,{d}^{3}p{d}^{3}p{\prime} {e}^{-i{\bf{p}}{\prime} \cdot {\bf{x}}}{e}^{i{\bf{p}}\cdot {\bf{x}}}[{\psi }_{FW}^{\dagger }({\bf{p}}{\prime} ){U}_{FW}({\bf{p}}{\prime} ){\bf{p}}{\prime} {U}_{FW}^{\dagger }({\bf{p}}){\psi }_{FW}({\bf{p}})\\  &  & +\,{\psi }_{FW}^{\dagger }({\bf{p}}{\prime} ){U}_{FW}({\bf{p}}{\prime} ){\bf{p}}{U}_{FW}^{\dagger }({\bf{p}}){\psi }_{FW}({\bf{p}})]\\  & = & \frac{1}{4mE(E+m)}\,\int \,{d}^{3}p{d}^{3}p{\prime} {e}^{-i{\bf{p}}{\prime} \cdot {\bf{x}}}{\psi }_{FW}^{\dagger }({\bf{p}}{\prime} )({\bf{p}}+{\bf{p}}{\prime} )\\  &  & \times \,[{(E+m)}^{2}+{\bf{p}}{\prime} \cdot {\bf{p}}-i{\bf{p}}{\prime} \cdot \Sigma \times {\bf{p}}]{e}^{i{\bf{p}}\cdot {\bf{x}}}{\psi }_{FW}({\bf{p}})\\  & \approx  & {\rho }^{2|l|-2}{\left(\frac{{d}_{0}(l)}{{w}^{|l|+1}}\right)}^{2}\left(\,-\,\frac{ly}{m},\frac{lx}{m},\frac{{p}_{0}}{m}{\rho }^{2}\right).\end{array}$$

Here we used the following calculations

50 $$\begin{array}{l}1.\,\int \,{d}^{3}p{d}^{3}{\bf{p}}{\prime} {e}^{-i{\bf{p}}{\prime} \cdot {\bf{x}}}{\psi }_{FW}^{\dagger }({\bf{p}}{\prime} )[(p+{\bf{p}}{\prime} )({(E+m)}^{2}+{\bf{p}}{\prime} \cdot {\bf{p}}){e}^{i{\bf{p}}\cdot {\bf{x}}}{\psi }_{FW}({\bf{p}})\\ \begin{array}{rcl} & = & {(E+m)}^{2}(i\nabla {\psi }_{FW}^{\dagger }({\bf{x}}){\psi }_{FW}({\bf{x}})-i{\psi }_{FW}^{\dagger }({\bf{x}})\nabla {\psi }_{FW}({\bf{x}}))\\  &  & -\,i\nabla {\psi }_{FW}^{\dagger }({\bf{x}})\cdot \nabla \nabla {\psi }_{FW}({\bf{x}})+i\nabla \nabla {\psi }_{FW}^{\dagger }({\bf{x}})\cdot \nabla {\psi }_{FW}({\bf{x}}).\end{array}\end{array}$$

The *x*-component of the above term for the LG solution becomes

51 $$\begin{array}{l}i{\partial }_{x}{\psi }_{FW}^{\dagger }({\bf{x}}){\psi }_{FW}({\bf{x}})-i{\psi }_{FW}^{\dagger }({\bf{x}}){\partial }_{x}{\psi }_{FW}({\bf{x}})\\ \,-i\nabla {\psi }_{FW}^{\dagger }({\bf{x}})\cdot \nabla {\partial }_{x}{\psi }_{FW}({\bf{x}})+i{\partial }_{x}\nabla {\psi }_{FW}^{\dagger }({\bf{x}})\cdot \nabla {\psi }_{FW}({\bf{x}})\\ \begin{array}{rcl} & = & {\left(\frac{{d}_{0}(l)}{{w}^{|l|+1}}\right)}^{2}{\rho }^{2|l|-4}[-2ly{(E+m)}^{2}{\rho }^{2}-4i{l}^{2}(|l|-1)y-2l{({p}_{0})}^{2}y{\rho }^{2}]\\  & \approx  & -4E(E+m)|l|y{\left(\frac{{d}_{0}(l)}{{w}^{|l|+1}}\right)}^{2}{\rho }^{2|l|-2}\end{array}\end{array}$$

using the physical vortex region condition of $\rho$. The $y$ and $z$ components are similarly calculated. And

52 $$\begin{array}{l}2.\,-\,i\,\int \,{d}^{3}p{d}^{3}p{\prime} {e}^{-i{\bf{p}}{\prime} \cdot {\bf{x}}}{e}^{i{\bf{p}}\cdot {\bf{x}}}{\psi }_{FW}^{\dagger }({\bf{p}}{\prime} )[({\bf{p}}+{\bf{p}}{\prime} ){\bf{p}}{\prime} \cdot \Sigma \times {\bf{p}}]{\psi }_{FW}(p)\\ \begin{array}{rcl} & = & -\,\nabla {\psi }_{FW}^{\dagger }({\bf{x}})\cdot \Sigma \times \nabla \nabla {\psi }_{FW}({\bf{x}})+\nabla \nabla {\psi }_{FW}^{\dagger }({\bf{x}})\cdot \Sigma \times \nabla {\psi }_{FW}({\bf{x}}).\end{array}\end{array}$$

The $x$-component of the above term (for $l > 0$) becomes

53 $$\begin{array}{l}-\,\nabla {\psi }_{FW}^{\dagger }({\bf{x}})\cdot \Sigma \times \nabla {\partial }_{x}{\psi }_{FW}({\bf{x}})+{\partial }_{x}\nabla {\psi }_{FW}^{\dagger }({\bf{x}})\cdot \Sigma \times \nabla {\psi }_{FW}({\bf{x}})\\ \begin{array}{rcl} & = & \langle {\Sigma }^{x}\rangle {\left(\frac{{d}_{0}(l)}{{w}^{|l|+1}}\right)}^{2}{\rho }^{2l-2}[\,-\,2l(l-1){p}_{0}({\cos }^{2}\,\phi -{\sin }^{2}\,\phi )+2{p}_{0}{l}^{2}{\rho }^{2l-2}]\\  &  & -\,\langle {\Sigma }^{y}\rangle {\left(\frac{{d}_{0}(l)}{{w}^{|l|+1}}\right)}^{2}{\rho }^{2l-2}4l(l-1){p}_{0}\,\cos \,\phi \,\sin \,\phi \\  &  & +\,4{l}^{2}(l-1)\langle {\Sigma }^{z}\rangle {\left(\frac{{d}_{0}(l)}{{w}^{|l|+1}}\right)}^{2}{\rho }^{2l-3}\,\sin \,\phi .\end{array}\end{array}$$

This term can also be ignored for the physical vortex region, when compared to the term in Eq. (51) with order $E(E+m){\rho }^{2l-1}$ because this term is order of ${p}_{0}{\rho }^{2l-2}$. The $y$ and $z$ term are similarly calculated.

Next let us calculate the numerator of the Dirac velocity:

54 $$\begin{array}{rcl}{\psi }^{\dagger }({\bf{x}})\alpha \psi ({\bf{x}}) & = & \int \,{d}^{3}p{d}^{3}p{\prime} {e}^{-i{\bf{p}}{\prime} \cdot {\bf{x}}}{e}^{i{\bf{p}}\cdot {\bf{x}}}\frac{E}{m}{\psi }_{FW}^{\dagger }({\bf{p}}{\prime} )\\  &  & \times \,{U}_{FW}({\bf{p}}{\prime} )\alpha {U}_{FW}^{\dagger }({\bf{p}}){\psi }_{FW}({\bf{p}})\\  & = & \frac{1}{2m}[i\nabla {\psi }_{FW}^{\dagger }({\bf{x}}){\psi }_{FW}({\bf{x}})-{\psi }_{FW}^{\dagger }({\bf{x}})i\nabla {\psi }_{FW}({\bf{x}})\\  &  & +\,\nabla \times ({\psi }_{FW}^{\dagger }({\bf{x}})\Sigma {\psi }_{FW}({\bf{x}}))]\end{array}$$

using only non-vanishing term $\beta (E+m)[\alpha \cdot {\bf{p}}{\prime} \alpha +\alpha \alpha \cdot {\bf{p}}]$ from $(E+m+\beta \alpha \cdot {\bf{p}}{\prime} )\alpha (E+m-\beta \alpha \cdot {\bf{p}})$. The first term that is equal to the FW velocity in the FW representation becomes

55 $$\begin{array}{c}\frac{1}{2m}[i\nabla {\psi }_{FW}^{\dagger }({\bf{x}}){\psi }_{FW}({\bf{x}})-{\psi }_{FW}^{\dagger }({\bf{x}})i\nabla {\psi }_{FW}({\bf{x}})]\\ \,\approx \,{\left(\frac{{d}_{0}(l)}{{w}^{|l|+1}}\right)}^{2}{\rho }^{2|l|-2}\left(-\frac{ly}{m},\frac{lx}{m},\frac{{p}_{0}}{m}{\left(\frac{{d}_{0}(l)}{{w}^{|l|+1}}\right)}^{2}{\rho }^{2}\right).\end{array}$$

And the 2*m*(second term) becomes

56 $$\begin{array}{c}\nabla \times ({\psi }_{FW}^{\dagger }({\bf{x}})\Sigma {\psi }_{FW}({\bf{x}}))\\ \,=\,{\left(\frac{{d}_{0}(l)}{{w}^{|l|+1}}\right)}^{2}{\rho }^{2|l|-2}(2|l|y\langle {\Sigma }^{z}\rangle ,-\,2|l|x\langle {\Sigma }^{z}\rangle ,2|l|(-y\langle {\Sigma }^{x}\rangle +x\langle {\Sigma }^{y}\rangle )).\end{array}$$

Note that ${\partial }_{z}{\psi }_{FW}^{\dagger }({\bf{x}}){\Sigma }^{x,y}{\psi }_{FW}({\bf{x}})=0$. Finally we obtain the Dirac velocity in Eq. (37).

### The expectation value of the *z* TEXT-component of the operator r × *α*

The expectation value of the *z*-component of the operator **r** × *α* is calculated as follows

57 $$\begin{array}{rcl}\langle x{\alpha }^{y}-y{\alpha }^{x}\rangle  & = & \int \,{d}^{3}x{\psi }^{\dagger }({\bf{x}})(x{\alpha }^{y}-y{\alpha }^{x})\psi ({\bf{x}})\\  & = & \int \,{d}^{3}p{\psi }_{FW}^{\dagger }({\bf{p}}){U}_{FW}({\bf{p}})(x{\alpha }^{y}-y{\alpha }^{x}){U}_{FW}^{\dagger }({\bf{p}}){\psi }_{FW}({\bf{p}}).\end{array}$$

The non-zero contributing terms of ${U}_{FW}({\bf{p}})(x{\alpha }^{y}-y{\alpha }^{x}){U}_{FW}^{\dagger }({\bf{p}})$ are

$$\begin{array}{l}\begin{array}{l}i\beta \frac{1}{2E}[{\alpha }^{y}\frac{{p}^{x}}{E}\frac{2E+m}{E(E+m)}\alpha \cdot {\bf{p}}+{\alpha }^{y}{\alpha }^{x}-i{\alpha }^{y}\alpha \cdot {\bf{p}}x\\ \,-\,\frac{2E+m}{E(E+m)}\frac{{\alpha }^{x}{p}^{y}}{E}\alpha \cdot {\bf{p}}-{\alpha }^{x}{\alpha }^{y}+i{\alpha }^{x}\alpha \cdot {\bf{p}}y]\\ \,+\,\frac{i\beta \alpha \cdot {\bf{p}}}{2E(E+m)}[\frac{2E+m}{{E}^{2}}{\alpha }^{y}{p}^{x}+\frac{{\alpha }^{y}{p}^{x}}{E}-i(E+m){\alpha }^{y}x\\ \,-\,\frac{2E+m}{{E}^{2}}{\alpha }^{x}{p}^{y}-\frac{{\alpha }^{x}{p}^{y}}{E}+i(E+m){\alpha }^{x}y]\end{array}\\ \begin{array}{rcl} & = & \beta \left[\frac{x{p}^{y}-y{p}^{x}}{E}+\frac{{\Sigma }^{z}}{E}+\frac{\Sigma \cdot {\bf{p}}{p}^{z}-{\Sigma }^{z}{\bf{p}}\cdot {\bf{p}}}{{E}^{2}(E+m)}\right].\end{array}\end{array}$$

Hence the expectation value of ${({\bf{r}}\times \alpha )}^{z}$ becomes $\frac{1}{E}(l+2\langle {S}_{D}^{z}\rangle +2(\langle {{\bf{S}}}_{D}\rangle \cdot \langle {\bf{p}}{p}^{z}\rangle -\langle {S}_{D}^{z}\rangle \langle {\bf{p}}\cdot {\bf{p}}\rangle )$/$(E(E+m)))$, which is twice of Eq. (20) of ref. ^10^.

Note that the above gives $\frac{1}{E}(l+2\langle {S}_{D}^{z}\rangle )$ for the paraxial approximation. However, the expectation value at **x** gives the Dirac spin-orbit interaction effect from Σ · **p***p*^*z*^ − Σ^*z*^**p** · **p** even though the average value of ${p}^{x}$ and ${p}^{y}$ over all space are zero for the paraxial LG solution in Eq. (47), because ${\psi }_{FW}^{\dagger }(x){p}^{x,y}{\psi }_{FW}(x)\ne 0$.
